# Histology of the tricuspid valve annulus and right atrioventricular muscle distance

**DOI:** 10.1093/icvts/ivac175

**Published:** 2022-07-08

**Authors:** Kokoro Yamane, Yosuke Takahashi, Hiromichi Fujii, Akimasa Morisaki, Yoshito Sakon, Noriaki Kishimoto, Takumi Kawase, Masahiko Ohsawa, Toshihiko Shibata

**Affiliations:** Department of Cardiovascular Surgery, Osaka City University Graduate School of Medicine, Osaka, Japan; Department of Cardiovascular Surgery, Osaka City University Graduate School of Medicine, Osaka, Japan; Department of Cardiovascular Surgery, Osaka City University Graduate School of Medicine, Osaka, Japan; Department of Cardiovascular Surgery, Osaka City University Graduate School of Medicine, Osaka, Japan; Department of Cardiovascular Surgery, Osaka City University Graduate School of Medicine, Osaka, Japan; Department of Cardiovascular Surgery, Osaka City University Graduate School of Medicine, Osaka, Japan; Department of Cardiovascular Surgery, Osaka City University Graduate School of Medicine, Osaka, Japan; Department of Diagnostic Pathology, Osaka City University Graduate School of Medicine, Osaka, Japan; Department of Cardiovascular Surgery, Osaka City University Graduate School of Medicine, Osaka, Japan

**Keywords:** Tricuspid annulus, Mitral annulus, Histology

## Abstract

**OBJECTIVES:**

Histologically, the mitral valve annulus comprises a collection of collagen fibres. However, the existence of collagen fibres in the tricuspid valve annulus has not been elucidated. Our goal was to clarify the histology of the tricuspid annulus.

**METHODS:**

Fifty human hearts without heart disease that were autopsied at Osaka City University Hospital between January 2009 and December 2017 were examined. The tricuspid valve was sectioned at 12 sites around the annulus, and the atrioventricular junction distance was measured.

**RESULTS:**

None of the tricuspid valve annulus samples had a continuous aggregation of collagen fibres that could be called an annulus. The interventricular space between the right atria and ventricles was composed of adipose tissue only on the anterosuperior and inferior sides, and no adipose tissue was found on the septal side. Comparing the atrioventricular muscle distance of the anterosuperior and inferior sides, the distance at the inferior side was statistically significantly larger than that of the anterosuperior side in 47 cases (*P *<* *0.0001).

**CONCLUSIONS:**

There was no continuous circumferential aggregation of collagen fibres in the right atrioventricular junction. The distance between the right atrial and ventricular myocardium was greater at the inferior side than that at the anterosuperior side, which might lead to more inferior annular dilation versus anterosuperior dilation. These anatomical features will be fundamental for future discussions of the suturing method used in prosthetic ring annuloplasty for tricuspid regurgitation.

**Figure:**
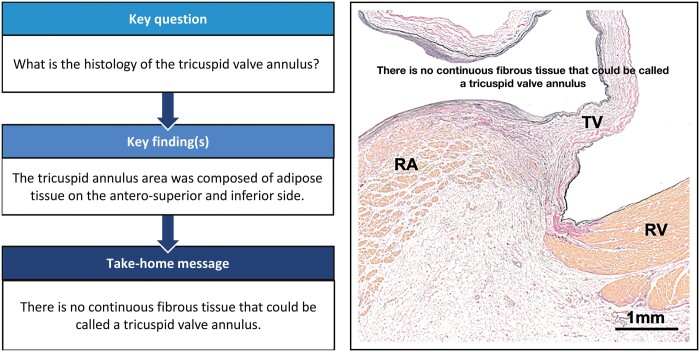

## INTRODUCTION

In mitral and tricuspid valve repair, a prosthetic annuloplasty ring is usually used. Therefore, it is important to understand the structure of the valve annulus. The anatomy and histology of the mitral valve annulus have been described previously [1–5]. The histological composition of the mitral valve annulus is not a string-like structure but a discontinuous fibrous structure that connects the left atrial wall to the left ventricular wall [1]. It is exceedingly rare to find a complete fibrous ring surrounding and supporting the leaflets [2, 3, 6].

Interest in the tricuspid valve annulus has increased in recent years. The analysis of tricuspid valve structure and morphological changes in the valve annulus in tricuspid regurgitation (TR) using echocardiography have been reported [7, 8]. In anatomical textbooks, the tricuspid valve annulus is described as less developed than the mitral valve annulus [9], and it is common for surgeons to have the impression that the tricuspid valve annulus tissue is fragile during tricuspid valve surgery. However, the actual histological composition of the tricuspid valve annulus has not been examined in detail to date. The purpose of this study was to examine the histology of the tricuspid valve annulus using autopsied hearts to clarify the structure of the annulus and its anatomical relationships with the right atrium and right ventricle.

## MATERIAL AND METHODS

### Ethical statement

Comprehensive written consent for histological studies using heart specimens was obtained in each case from the patients’ families when the pathological autopsies were performed. This study was approved by our institutional review board on 30 September 2016 (approval number: 3555).

### Methods

This study was performed using 50 autopsy hearts obtained from Osaka City University Hospital from January 2009 to December 2017. The 50 cases were defined as those in which the tricuspid valve annulus, including the right atrium, right ventricle and tricuspid valve, could be observed circumferentially, with no cardiac disease (valvular disease, coronary artery disease and arrhythmia), and whose tricuspid valve consisted of 1 anterosuperior, 1 inferior and 1 septal leaflet. Patient information, including medical history, echocardiographic findings and cause of death were obtained from the donors’ medical records.

**Figure 1:**
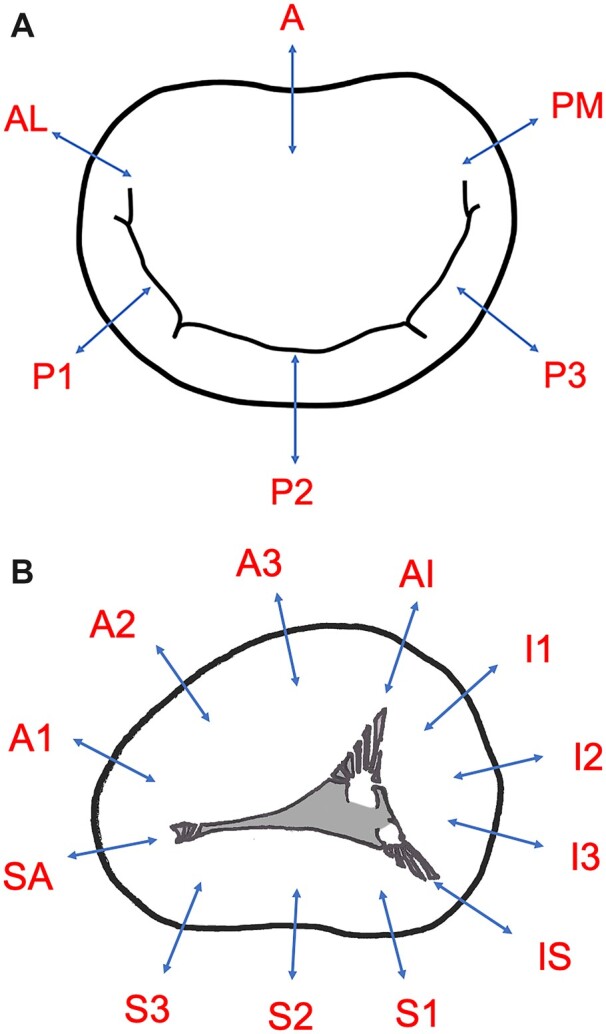
Preparation of the sections. (**A**) The mitral annulus was divided into 6 sections. A is the midpoint of the annulus on the anterior leaflet side; AL is the anterolateral commissure; PM is the posteromedial commissure; and P1-P3 are the midpoints of each posterior leaflet. AL: anterolateral; PM posteromedial. (**B**) The tricuspid annulus was divided into 12 sections as follows: each commissure (AI, IS, SA) and the quadrants between the commissures (A1–3, I1–3, S1–3). AI: anterosuperior–inferior; IS: inferior–septal; SA: septal–anterosuperior.

At the pathological autopsy, the hearts were removed and fixed using formalin solution. For the mitral valves, sections were made the midpoint of the annulus on the anterior side (A), the anterolateral commissure (AL), the posteromedial commissure (PM) and the midpoint of each posterior leaflet (P1, P2, P3) (Fig. 1A). For the tricuspid valve, the right atrium and right ventricle were cut open at the commissure of the inferior and septal leaflets. Sections were made at 3 locations on each commissure and 9 locations where each leaflet’s annulus was divided into 4 equal parts. The commissures between the anterosuperior and inferior leaflets, between the inferior and septal leaflets, and between the anterosuperior and septal leaflets were designated AI, IS, and SA respectively. The commissures where the anterosuperior leaflet annulus was divided into 4 equal parts were designated as A1–A3 from the septal side; where the inferior leaflet annulus was divided into 4 equal parts as I1–I3 from the anterosuperior leaflet side; and where the septal leaflet annulus was divided into four equal parts as S1–S3 from the inferior leaflet side (Fig. 1B). Sections were made perpendicular to the valve annulus to include the atrial muscles, tricuspid valve and ventricular muscles, and hematoxylin and eosin and elastic van Gieson stains were used. In each section, the presence or absence of collagen fibre aggregation at the valve attachment area and the relationships between the atrial muscles, ventricular muscles and valve leaflets were observed in detail. The elastic van Gieson-stained sections were examined using a light microscope under 40× magnification to measure the distance between the edge of the right atrial muscle and the edge of the right ventricular muscle (atrioventricular muscle distance: AVMD).

### Statistical analysis

The mean AVMD (µm) was compared for each section, including the anterosuperior and inferior sides, using Wilcoxon’s signed rank sum test. JMP version 16.0 (SAS Institute Inc., Cary, NC, USA) was used for statistical analysis, and the significance level was set at less than 5%.

## RESULTS

### Patient background

The age of the patients ranged from 22 to 89 years [median (standard deviation): 62.3 (13.7) years]; 30 patients (60%) were male, and 20 patients (40%) were female. The cause of death was malignancy in 26 cases (52%), infection in 10 cases (20%), neurological disease in 5 cases (10%) and other in 9 cases (18%) (Table 1).

**Table 1:** Characteristics of the patients providing the autopsy hearts

Characteristic		Patient numbers (N = 50)
Age, years; mean (SD)		62.3 (13.7)
Sex	Male	30 (60%)
	Female	20 (40%)
Cause of death	Malignant disease	26 (52%)
	Infection	10 (20%)
	Neurological disease	5 (10%)
	Other	9 (18%)

SD: standard deviation

### Histology of the mitral and tricuspid valve annuli

**Figure 2:**
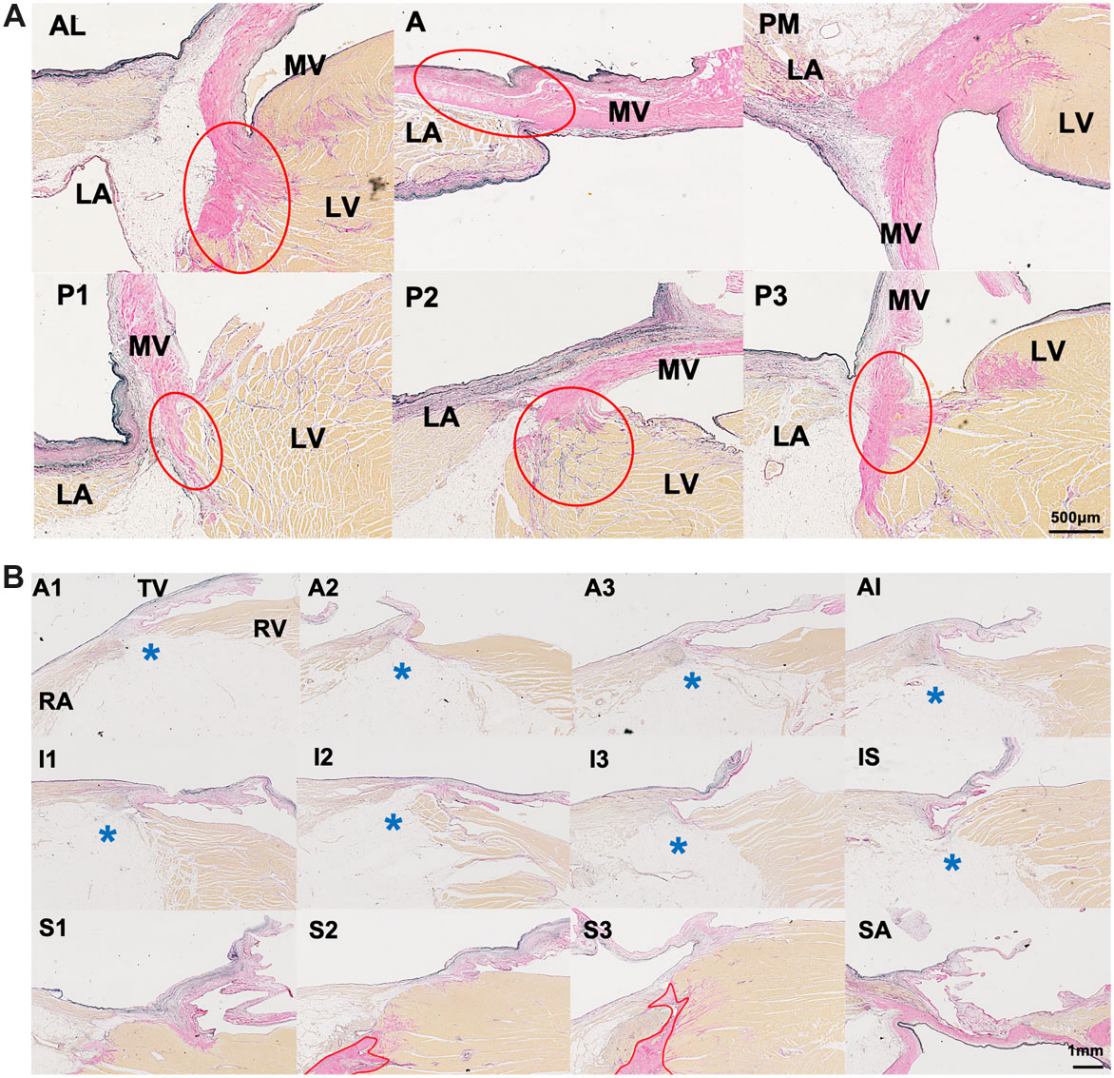
Histology of the mitral and tricuspid valve annuli. (**A**) Six sections (A/AL/PM/P1-P3) of a typical mitral annulus in a representative case. The red circles are aggregations of collagen fibres, which are visible in all sections. On the AL site, fibrous tissue is present but not between the atrioventricular muscles, and on the P1 site, fibrous tissue can be seen like a curtain. Elastic van Gieson (EVG) staining; magnification ×20. Scale bar indicates 500 µm. AL: anterolateral; LA: left atrium; LV: left ventricle; MV: mitral valve; PM: posteromedial. (**B**) Twelve sections (A1–SA) of a typical tricuspid annulus in a representative case. No circumferential tissue characteristic of a valve annulus, such as collagen fibre aggregation, was observed. The asterisk indicates adipose tissue. The area within the red circle is the central fibrous body. Scale bar indicates 1 mm. EVG staining; magnification ×20. A: anterior; RA: right atrium; RV: right ventricle; S: septal; TV: tricuspid valve.

**Figure 3:**
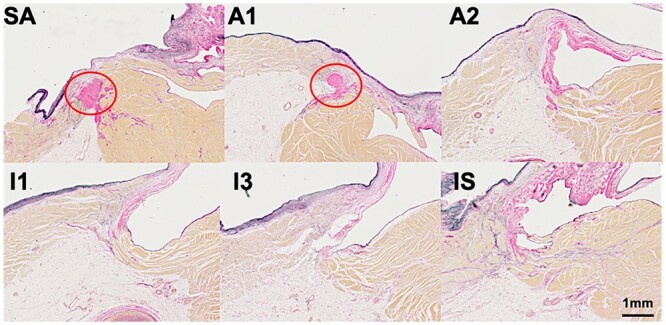
Partial aggregation of collagen fibres. A case of localized collagen fibre aggregation (red circle) at the valve attachment only in the SA and A1 sections. Elastic van Gieson staining; magnification: ×20. Scale bar indicates 1 mm. A: anterosuperior; SA: septal–anterosuperior.

In the mitral valve annulus, fibrous tissue consisting of an aggregation of collagen fibres was observed. At some point, the fibrous tissue was cord-like or curtain-like and varied in thickness depending on the site (Fig. 2A). It was possible to find areas with no fibrous tissue supporting the mural leaflet. In the tricuspid annulus, there was no circumferential aggregation of collagen fibres at the right atrioventricular area in any of the 50 cases (Fig. 2B). In 9 of the 50 cases, a partial aggregation of collagen fibres was observed in the atrioventricular area as follows: in segment SA in 3 of 50 cases, in A1 in 8 cases, A2 in 3 cases and A3 in 1 case. All aggregations were localized to only 1 or 2 of the 12 sections and did not comprise continuous circumferential collagen fibre tissue. A case of localized collagen fibre at SA, A1, A2 and A3 is shown in Fig. 3.

### Tissue between the atrial and ventricular muscles

**Figure 4:**
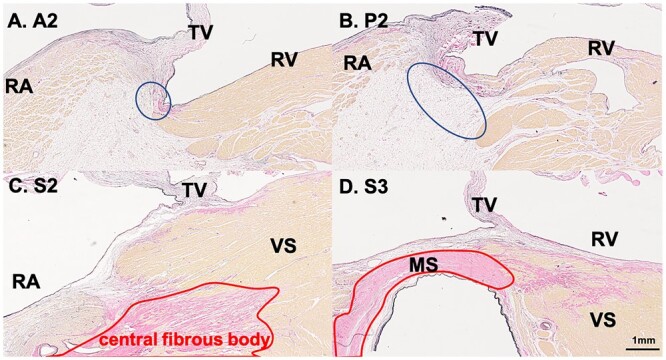
Histology of the right atrioventricular area. (**A, B**) The area between the atrial and ventricular muscles at the anterosuperior and inferior sides is composed of adipose tissue (blue circles). Sections are A2 (**A**) and I2 (**B**). (**C, D**) The area between the atria and ventricle at the septal side is connected via a central fibrous body (outlined in red), without intervening adipose tissue. Sections are S2 (**C**) and S3 (**D**), and (**D**) is at the site of the membranous septum. A: anterosuperior; I: inferior; MS: membranous septum; RA: right atrium; RV: right ventricle; S: septal; TV: tricuspid valve; VS: ventricular septum.

Histologically, most of the area between the atria and ventricles at the attachment of the anterosuperior and inferior leaflets was composed of adipose tissue (Fig. 4A, B). In contrast, the area between the atria and ventricle at the septal side was connected via a central fibrous body containing a membranous septum, with little or no adipose tissue (Fig. 4C, D).

### Atrioventricular muscle distance

**Figure 5:**
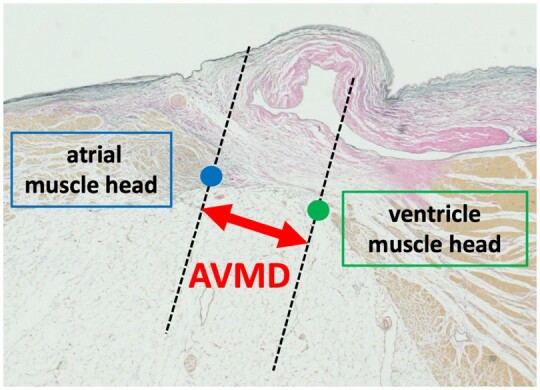
Right atrioventricular muscle distance. The edges of the right atrial muscle (blue dot) and right ventricular muscle (green dot) were identified, and the distance (µm) between these muscles was measured as the atrioventricular muscle distance (AVMD; red arrow).

**Figure 6:**
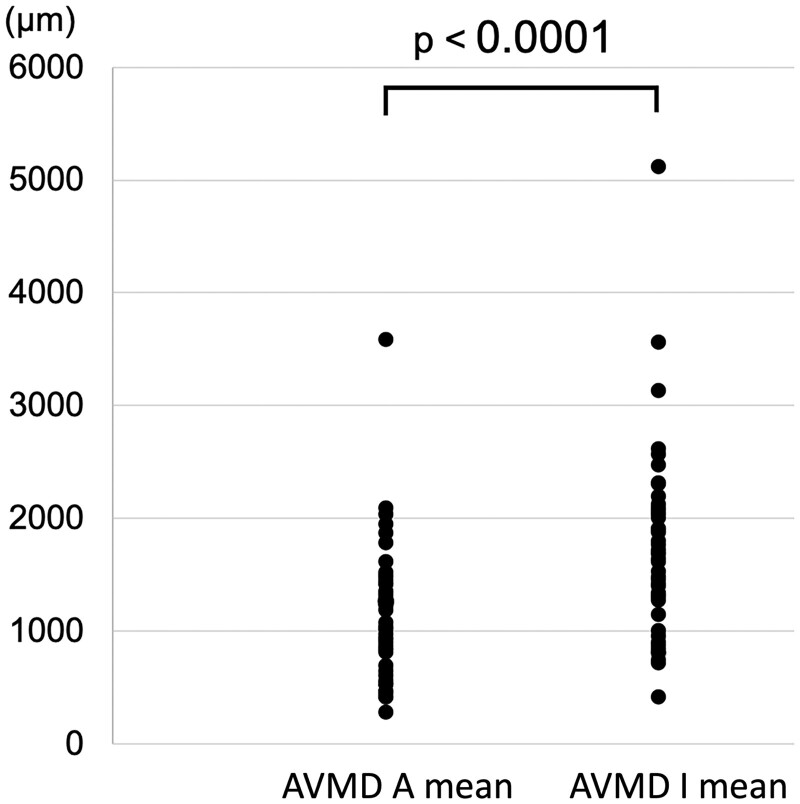
Comparison of the anterosuperior AVMD and inferior AVMD. Comparison of the mean anterosuperior AVMD and the mean inferior AVMD in each case. Wilcoxon’s signed rank sum test was performed using the difference between the mean AVMD values, which showed that the mean AVMD at the inferior side was larger than that at the anterosuperior side (*P* < 0.0001). A: anterosuperior; AVMD: atrioventricular muscle distance; I: inferior.

The AVMD (µm) in each section of the anterosuperior and inferior sides (A1–3, I1–3) is shown in Fig. 5. In 47 (94%) of 50 cases, the mean AVMD at the inferior side was longer than that at the anterosuperior side. The mean AVMD was significantly larger at the inferior side [1629 (1244–2060) µm] compared with the anterosuperior side [1059 (688–1425) µm; (*P *<* *0.0001] (Fig. 6).

## DISCUSSION

### Histology of the tricuspid valve annulus

In this study of 50 autopsied hearts, we showed that the tricuspid valve has no circumferential collagen fibres, which means that a tricuspid valve annulus does not exist.

In 1980, Anderson *et al.* reported that the tricuspid annulus was far less well formed than the mitral annulus and was composed mostly of conjoined valve leaflets, without a complete thickened collagenous ring [1]. In 1985, Wilcox *et al.* reported that a histological section across the right atrioventricular area showed that it was the fibrofatty tissue of the atrioventricular groove and that there was no fibrous “annulus” supporting the hinge of the tricuspid valve [10]. Since then, few histological studies of the tricuspid valve annulus have been performed. In 2012, Messer *et al.* examined the tissue of the tricuspid valve annulus using 12 autopsied hearts. The authors reported that there was minimal fibrous tissue on the anterosuperior and inferior sides [11]. These results are consistent with the findings in our study. In the present study, we included more cases and studied these in detail by examining the histological characteristics of the right atrioventricular area, the tissue between the atrial and ventricular muscles and the AVMD.

Based on the anatomical position of the tricuspid valve, it is composed of anterosuperior, inferior and septal leaflets. On the other hand, anterosuperior leaflets are described as anterior leaflets and inferior leaflets and as posterior leaflets in surgical and clinical settings. Victor and Nayak divided the tricuspid valve in 2 parts as a mural leaflet (anterosuperior and inferior leaflet) and a septal leaflet [12]. Histologically, there is a noticeable difference between the anterosuperior, inferior side and the septal side. Generally, it has been reported that tricuspid annular dilatation occurs at the anterosuperior and inferior sides [7, 13]. Our study revealed the absence of continuous fibrous tissue in the right atrioventricular junction on the anterosuperior and inferior sides, with some fibrous tissue on the septal side. This result was considered histologically consistent with the fact that, according to previous reports, the anterosuperior and inferior annuli tend to expand under pressure and under volume loading [4, 12].

The present study also showed that (i) there was a partial aggregation of fibrous tissue in the anterosuperior leaflet in 9 of 50 cases and that (ii) when the anterosuperior and inferior annuli were compared regarding AVMD, the distance at the inferior side tended to be greater than that on the anterosuperior side. These histological results support the theory that tricuspid valve annulus enlargement is more likely to occur on the inferior leaflet side. TR progresses easily when right atrial enlargement and inferior annular dilation occur because of atrial fibrillation [14, 15]. This mechanism also appears to be supported by our previous histological results. In that report, only 1 case with atrial fibrillation, severe tricuspid regurgitation and enlarged tricuspid valve leaflets was studied by making sections using the same method as that used in the present study. No fibrous tissue in the valve annular area on the anterosuperior and inferior leaflets, and only adipose tissue between the atrioventricular muscles, were observed. However, there was a difference in the AVMD. The mean AVMD at the anterosuperior side was 2293 µm, and the mean AVMD at the inferior side was 8500 µm, indicating considerable enlargement compared with the AVMD values in the current study. A relationship between increased AVMD and annulus enlargement is possible, especially in the inferior annular area in patients with TR.

In 1988, Hutchins *et al.* defined disjunction as a separation of the atrial and ventricular myocardial segments in the mitral valve and described the relationship between mitral regurgitation and disjunction [4]. Furthermore, Wells and Anderson *et al.* showed that the relationship with mitral valve disjunction was also seen in normal hearts [6]. Surprisingly, in the present study, no fibrous tissue or the existence of an AVJ was observed on the anterosuperior and inferior sides, even in the absence of a medical history of heart disease. These results mean that there might be a disjunction in the anterosuperior and inferior tricuspid annular area. However, we did not examine the relationship between disjunction and arrhythmia and TR: Further research is needed.

The limitations of this study are as follows: First, the mean age of the autopsy cases was >65 years, and the influence of histological changes related to ageing may not have been eliminated. It will be necessary to examine changes related to ageing in a future study. Second, only patients without a history of cardiac disease were studied, and the results were not compared with those of patients with cardiac disease, especially with tricuspid valve disease and tricuspid valve annulus enlargement. However, we speculate that there may be a relationship between increased AVMD and posterior annulus enlargement. Future studies in patients with significant TR and tricuspid valve annular enlargement are warranted.

## CONCLUSION

In the right atrioventricular area, there was no circumferential aggregation of collagen fibres that could be generally considered annular tissue. In addition, the distance between the right atrial and right ventricular myocardium tended to be greater on the inferior leaflet side compared with the anterosuperior leaflet side.

## Data Availability

All data are incorporated into the article and its online supplementary material.

## ABBREVIATIONS

AVMD: Atrioventricular muscle distance
TR: Tricuspid valve regurgitation
